# Evaluation of the Early Development of 6-Month-Old Babies in the Case of Maternal Postpartum Depression with or Without Bipolar Disorder

**DOI:** 10.3390/children12010011

**Published:** 2024-12-24

**Authors:** Jokthan Guivarch, Mélanie-Lou Persia, Laure Le Treut, Pauline Grandgeorge, Federico Solla, Hugo Pergeline, Michel Dugnat, Florence Askenazy, François Poinso, Arthur Varoquaux, Arnaud Fernandez

**Affiliations:** 1 Faculty of Medicine, Aix-Marseille University, 13385 Marseille, France; jokthan.guivarch@ap-hm.fr (J.G.); hugo.pergeline@ap-hm.fr (H.P.); francois.poinso@ap-hm.fr (F.P.); arthur.varoquaux@ap-hm.fr (A.V.); 2 Department of Child Psychiatry, Assistance Publique Hôpitaux de Marseille (APHM), 13009 Marseille, France; melanie.persia@ap-hm.fr (M.-L.P.); pauline.grandgeorge@ap-hm.fr (P.G.); michel.dugnat@ap-hm.fr (M.D.); 3 Institut de Neurosciences de la Timone, UMR 7289, Centre National de la Recherche Scientifique (CNRS), Aix-Marseille University, 13385 Marseille, France; 4 Department of Child Psychiatry, Valvert Hospital, 13011 Marseille, France; laure.letreut@ch-valvert.fr; 5 Pediatric Surgery Department, Lenval University Children’s Hospital, 57 Bd Californie, 06200 Nice, France; 6 Healthcare Department, Link Campus University, 00165 Rome, Italy; 7 Lenval University Children’s Hospital, SUPEA (University Department of Child and Adolescent Psychiatry), Competence Center for Rare Diseases with Psychiatric Expression (CC MREP), Expert Center for Pediatric Psychotrauma (CE2P), 06200 Nice, France; florence.askenazy@hpu.lenval.com (F.A.); arnaud.fernandez@hpu.lenval.com (A.F.); 8 EA CoBTek, Faculty of Medicine, University Côte d’Azur, 06100 Nice, France; 9 Department of Medical Imaging, Conception University Hospital, Aix-Marseille University, 13005 Marseille, France; 10 Centre de Résonnance Magnétique Biologique et Médicale-Centre d’Exploration Métabolique par Résonance Magnétique (CRMBM—CEMEREM) (UMR 7339), CNRS, La Timone Medical School, Aix-Marseille University, 27 Bd J. Moulin, 13385 Marseille, France

**Keywords:** postpartum depression, child development, development quotients, mother–baby unit, Brunet–Lézine scale, mother–infant interaction

## Abstract

Background: The first year of life is the period of greatest brain plasticity. Postpartum depression can adversely affect the first interactions with the child and, consequently, their emotional, social, and cognitive development. Objectives: First, to describe the developmental profile of six-month-old infants of mothers suffering from severe postpartum depression, and, second, to compare the development of infants whose mothers suffer from depression with or without bipolar disorder. Methods: This is a retrospective descriptive study on 6-month-old babies hospitalized with their mothers at the Marseille Mother–Baby Unit (MBU) for maternal postpartum depression with or without bipolar disorder. Mothers were clinically diagnosed by a psychiatrist specialized in postpartum depression using the DSM-5; infant development was assessed at 6 months by an independent health professional using the revised Brunet–Lézine Scale, which allowed the calculation of global and partial developmental quotients (DQ). Results: We followed 40 mother–infant dyads. None of the 40 infants had a global developmental delay. However, maternal depression was significantly associated with poorer sociability (mean sociability DQ score of 94 ± 9.6, *p* < 0.001) and lower postural development (mean postural DQ score of 96.2 ± 8.9 *, *p* < 0.001) in the infants at 6 months of age. Postural development was significantly lower in children of bipolar mothers than in children of non-bipolar mothers (*p* = 0.03). Conclusions: Postpartum depression was associated with a weakness in sociability and posture at the age of 6 months, without relevant developmental delay. Screening infants at an early age with specific tools allows for earlier intervention, which would positively influence their developmental trajectory.

## 1. Introduction

Postpartum depression (PPD) affects approximately 12% of new mothers in high-income countries [1]. A recent cohort study in France (IGEDEPP) suggests a prevalence of postpartum depression of about 8% at 8 weeks and 12.9% at 1 year [2]. PPD can have a negative impact on early mother–child interactions, which can subsequently affect the child’s emotional, social, and cognitive development [3].

Regarding the course of symptoms, it has been found that 30% of women who experience postpartum depression continue to be significantly affected more than one year after delivery [4]. Some authors argue that the prolonged and/or recurrent nature of the mood disorder may have an impact on the child’s developmental trajectory [5,6]. According to Murray [7], the early experience of a lack of maternal sensitivity in interaction is the most detrimental to infant development. Moreover, Stein [8] suggests that depression leads to a lack of maternal sensitivity, especially in socially disadvantaged women.

When the parents have disabling psychiatric disorders, Fraiberg suggests that it is necessary to objectively ensure the development of the infant in an objective manner [9]. To treat the effects of PPD on infants, early parent–child care has been developed in mother–baby units (MBUs) to treat both puerperal and maternal disorders, as well as difficulties in the mother–child relationship. These units were inspired by joint hospitalization practices in the United Kingdom, as described by Brockington and Kumar [10,11]. In France, 58% of hospital admissions to MBU occur in the first 8 weeks postpartum [12].

The symptomatic features of PPD differ from those of classic depression. Anxiety plays a central role in PPD and may mask the sadness of the mood [13]. This anxiety may be expressed as an impulse phobia, an excessive fear of harming the child, infant-centered worries focused on the infant outside any real pathological context, and intrusive obsessive thoughts [14]. PPD is characterized by feelings of discouragement and an inability to cope with motherhood.

The impact of maternal postpartum depression on the child is of current interest to researchers, as evidenced by several publications [15,16,17]. Some authors examine the impact of depression on attachment [16], on the child’s mental health [17], or attempt to quantify the strength of the association between maternal depression and emotional or behavioral disorders in children and adolescents [15]. Other authors have examined the effects of PPD on children’s behavior and long-term cognitive, social–emotional, and language development [18,19,20]. They have shown that the impact on long-term development is correlated with “prolonged maternal depressive symptoms” [18]. Authors suggest that postpartum psychiatric disorders affect early child development, “including neurosynaptic and regulatory developments and developmental milestones” [21]. PPD affecting the mother–child relationship, known as “attachment and bonding”, could create an environment that affects development and “neuronal migration, synapse formation, and pruning” [22].

However, there is a paucity of data on the early effects of PPD on infant development [18,23] and particularly few studies using a specific instrument to objectively assess the development of infants whose mothers suffer from severe depression. Exploring a baby’s development involves looking at different areas of development: motor skills (posture, walking, gross and fine motor skills), grasping and oculomotor coordination, sensorimotor and cognitive development, social–emotional development (recognizing emotions and developing relationships with others), and communication and language (preverbal, verbal, and nonverbal, receptive and expressive) [24,25,26,27].

PPD is a condition with clinical heterogeneity that allows the identification of different subgroups. These subgroups, or phenotypes, may affect the relationship with the child differently and thus have different effects on child development and the mother–child relationship [28]. Among these subgroups is bipolar disorder. We may wonder whether maternal PPD with bipolar features has a particular impact on the development of the baby. Bipolar depression is known to have higher morbidity and mortality rates and more relapses than non-bipolar depression. In addition, subsyndromal symptoms between episodes are observed in bipolar disorder, which contribute to the long-term social dysfunction of the person [29]. Anke et al. studied the interactional qualities of babies with bipolar mothers compared with those of babies with mothers without psychiatric disorders. The authors found a significant deterioration in the quality of mother–baby interactions when the mother had bipolar disorder, regardless of the mother’s symptom burden [30]. However, the authors did not measure the effect on infant development, nor did they compare the effect of bipolar depression with “common” PPD outside of bipolarity. Given the more pronounced functional impairment in depression associated with a bipolar disorder [29], the deficit in emotion identification and social cognition even in the euthymic period, and the more impaired quality of interpersonal relationships in bipolar disorder [31], it is reasonable to suspect a special impact of bipolar depression on the baby’s development. It is also important to consider bipolar disorder because of its high prevalence and frequent revelation during a postpartum depressive episode [32].

We hypothesized that the early development of infants of depressed mothers would be affected by postpartum depression, and that the effect would be greater if the mother suffered from bipolar disorder.

Our primary objective was to describe the developmental profile of six-month-old infants of mothers with severe PPD, in search of a very early effect of PPD on child development.

Our secondary objective was to compare the development of infants whose mothers suffer from depression evolving with or without a bipolar disorder.

## 2. Materials and Methods

### 2.1. Study Design

This is an exploratory, retrospective, descriptive study analyzing the development quotients (DQ) of infants at the age of 6 months who were jointly hospitalized with their mothers in a specialized unit for PPD.

### 2.2. Population

#### 2.2.1. Inclusion Criteria

This study included infants aged 6 ± 1 months who were hospitalized with their mothers for PPD between 1 January 2012 and 30 June 2023 at the Mother–Child Unit of the Sainte-Marguerite University Hospital in Marseille, France. Data analysis was performed in July 2023.

In France, MBUs are specialized in postpartum psychiatric disorders care for mother–baby dyads referred by obstetrics, psychiatry, child psychiatry, or pediatrics departments. These units care for mothers with severe postpartum psychiatric disorders that affect the mother–baby relationship and their babies. The goal of the care provided is to treat both the mother’s depression and the mother–child relationship. The intervention used is based on the continuous presence of psychiatric and pediatric nurses for mother and child, respectively. Such healthcare providers play a role in both prevention and early intervention in the child’s development. This is achieved without separating the dyad in order to prevent the onset of problems in the mother–child relationship that can lead to disorders in the child [12].

Our MBU is a weekday unit, meaning that the child and mother are hospitalized during the week and return home on weekends. This implies that, as a condition of admission to the unit, it is necessary to have an available entourage at home to help or substitute for the mother in her maternal caregiving functions when she is depressed, as the caregivers in the MBU do during the week. A second condition for admission to the unit is that there is no identified risk of danger to the mother or child, i.e., no high risk of suicide or infanticide.

#### 2.2.2. Non-Inclusion Criteria

Infants born before 35 weeks of gestation were not included.

#### 2.2.3. Exclusion Criteria

Infants with intrinsic features that could interfere with DQ results, such as neurodevelopmental disorders, sensory disorders, genetic syndromes, and neonatal cardiorespiratory arrest with risk of anoxic encephalopathy, were excluded. In addition, infants with ultrasound anomalies (IUGR, morphological abnormalities) or who had been exposed in utero to fetotoxic substances were excluded.

Finally, children whose mothers had psychotic symptoms were also excluded.

#### 2.2.4. Population Included

The study population consisted of all children who met the inclusion and non-inclusion criteria and who did not meet any exclusion criteria, without sampling.

### 2.3. Data Collected

Data were collected in the MBU on the primary endpoint, which included maternal sociodemographic characteristics, such as age and parental status (single mother or in a relationship), and medical characteristics, including diagnosis (PPD with or without bipolarity and with or without anxiety-related comorbidities), psychiatric history, parity (first child or not), and length of hospitalization. Diagnoses were based on clinical examination using DSM-5-TR criteria [33]. Sociodemographic characteristics and exclusion criteria were obtained from parental interviews and medical records.

### 2.4. Judging Criterion

The primary endpoint of the study was the Brunet–Lézine Development Quotient (DQ) [26].

Early childhood development can be assessed using several available scales. The Bayley Scales of Infant Development—3rd Edition (BSID-III) is the most widely used scale in the literature to assess the development of preschool children (between 1 and 42 months) [34]. It consists of five scales: cognitive, language, motor, social–emotional, and behavioral. The cognitive, language, and motor scales are scored by the examiner, while the social–emotional and behavioral scales are scored by the parents through a questionnaire [27]. Unfortunately, this instrument was not available in French before 2022. Therefore, it was not possible to use it to collect data on the babies included in our study, which started in 2012.

To assess early childhood development, we naturally used the revised Brunet–Lézine (BL) scale, which is the second most widely used scale and the main early childhood development scale used in France [35]. It assesses language, posture, coordination, and sociability in infants aged 2 to 30 months, with calculations of partial and global DQ. It provides an assessment at a given time of the child’s development: at 6 months, 10 items are tested (lying on the back grasps his feet with his hands, removing the towel placed on his head, rising up to a sitting position, holding two cubes in each hand…). By scoring the successes and failures of the tested items, we can place the child in age groups and calculate developmental scores. The partial and global DQ are obtained by relating the developmental age observed during the assessment (OA) to the child’s chronological age (CA) using the formula DQ = (OA/CA) × 100. Global and partial DQ scores follow a Gaussian curve with normal values between 80 and 120 and a mean of 100. Based on this scale, a developmental delay is defined as an overall DQ below 70 [26,36]. The examiner gathers information about the child’s abilities through observation and by interviewing the parents. This instrument has good psychometric properties for assessing the development of infants up to two years of age [26]. It has been validated on a French population of 1050 infants aged from 2 to 30 months, including 54 babies aged 6 months (27 boys and 27 girls).

The revised Brunet–Lézine (BL) scale is comparable to the Bayley Scales of Infant Development—3rd Edition (BSID-III) [27]. Indeed, a comparative study between the BL and the BSID-III showed moderate correlations between the gross motor score of the BSID-III and the posture quotient of the BL, between the fine motor score of the BSI-III and the hand–eye coordination quotient of the BL, and between the socio-emotional score of the BSID-III and the sociability quotient of the BL. It should be noted that the study was conducted on a population of 36 pre-term infants with a corrected age of 6 months. However, the BSID-III language subscore and the BL language quotient did not correlate well [37].

### 2.5. Course of the Study

#### 2.5.1. Diagnosis of Mothers

The diagnosis was made during the pre-admission consultation based on the DSM-5 criteria [33].

#### 2.5.2. Assessment of the Developmental Quotient (DQ)

The BL test is routinely performed on all infants hospitalized in our unit at 6, 12, and 18 months of age. The DQ assessment was conducted by a trained female psychologist who was external to our unit. In other words, she was not involved in the treatment of each dyad. Moreover, she was unaware of the mothers’ diagnoses and of the study’s aim.

### 2.6. Statistical Methods

Categorical variables were presented as numbers of patients with percentages and compared using the Fisher’s Exact Test. Continuous variables were expressed as means ± standard deviations (SD) or as medians with interquartile range (IQR). Normally distributed parameters, as defined by the Shapiro–Wilk test, were compared using Student’s *t*-test; nonparametric data were tested using the Wilcoxon signed rank test. Holm’s correction procedure was used to adjust the *p*-values in multiple hypothesis tests performed simultaneously. All tests were two-tailed, and *p* < 0.05 was considered to indicate a statistically significant difference. Statistical analyses were performed using R statistical software (R Foundation for Statistical Computing, Vienna, Austria, R version 4.3.3).

### 2.7. Research Procedures and Compliance with Ethical Standards

The parents of the children provided their informed consent for the use of their data, which were anonymized.

The Scientific and Ethical Committee of the APHM (Marseille, France) approved the study on 12 July 2023, registered under number CSE 23-4.

## 3. Results

### 3.1. Sample Characteristics

#### 3.1.1. Source Population

The initial study population included 48 infants who were 6 months old. A total of 5 infants out of 48 were excluded due to intrinsic factors: 2 with neonatal anoxic encephalopathy, 1 with clubfoot, 1 with microdeletion syndrome, and 1 exposed to fetotoxic products in utero.

**Figure 1:**
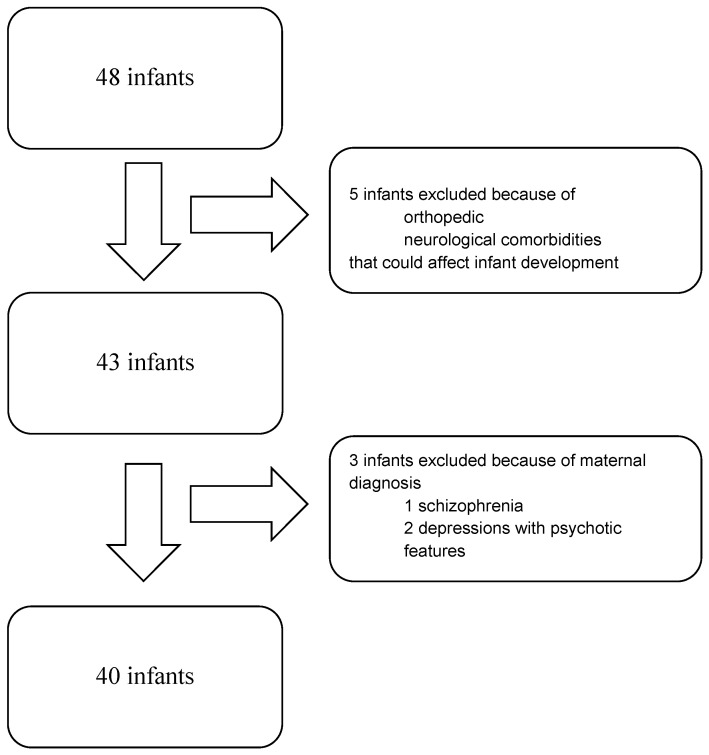
Flow chart of the study population.

Moreover, three infants were excluded because of maternal psychotic features. A total of 40 developmental quotients (DQs) from 40 patients were analyzed (Figure 1).

#### 3.1.2. Included Infant Population

The study population consisted of 40 infants, including 22 girls and 18 boys. Mean and median infant age was 6.4 [0.8] months, ranging from five months and two days to seven months.

#### 3.1.3. The Mothers

There were 38 mothers in the study, including two with twin pregnancies (Table 1). The median age of the participants was 33 [8.2] years (range: 20–46; mean age: 34). Three mothers were separated from the father of their child.

Of the 38 mothers, 28 were primiparous. A history of previous depression was reported by 21 mothers, including 8 with a known history of bipolar disorder. Two mothers were diagnosed with bipolar disorder while in the mother–child unit due to the occurrence of a manic or hypomanic episode. Ten mothers presented bipolar disorder at the admission. Moreover, 30 mothers had a comorbid anxiety disorder.

The median length of hospitalization was 170 (121) days (range: 19–506; mean length: 191.4). Hospitalizations were both conventional (overnight) or day, usually first conventional and then day.

Table 2 describes the medical and sociodemographic characteristics of the mothers, differentiated by the presence of bipolar disorder (Table 2).

### 3.2. Main Results

The global mean DQ score was 96.9 ± 6.7, with a median global score of 96.5 (*p* < 0.001) (min 78; max 119) [Q1 94–Q3 100].

Two partial DQs were statistically reduced compared to the 100 value: the mean posture DQ score, which was 96.2 ± 8.9 * (*p* < 0.001), and the mean sociability DQ score, which was 94 ± 9.6 (*p* < 0.001). Table 3 shows the main results of the developmental scale.

### 3.3. Secondary Outcomes

**Figure 2:**
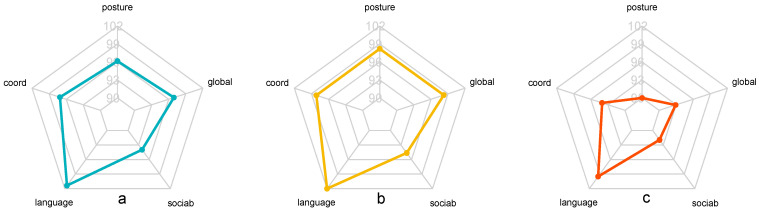
Developmental quotients (DQs) of babies according to whether or not the mother suffers from bipolarity. (**a**) DQ of babies of bipolar and non-bipolar mothers; (**b**) DQ of babies of non-bipolar mothers; (**c**) DQ of babies of bipolar mothers.

The postural partial quotient of infants born to bipolar mothers was significantly lower than that of infants born to non-bipolar mothers (*p* < 0.03). The global mean developmental quotient of infants born to bipolar mothers was not significantly reduced (Table 4 and Figure 2).

## 4. Discussion

### 4.1. Summary of Key Results

We aimed to assess the effects of PPD on the development of 6-month-old infants. Although the results showed a decrease in the overall DQ (*p* < 0.001), no developmental delay was found in any of the 40 babies. Maternal depression was associated with poorer sociability and lower postural development (*p* < 0.001). Postural development was lower in infants of bipolar mothers than in infants of non-bipolar mothers (*p* < 0.05).

### 4.2. Discussion of Results Regarding Population Characteristics

Most of the women in our cohort were first-time mothers, which is consistent with the existing literature [28,38].

Approximately half of the women had a history of psychiatric disorders, also consistent with previous research [28]. It is well known that women with a history of mood disorders are known to have a significantly higher risk of developing PPD [39], which may explain the high proportion of women with a psychiatric history in our cohort. Six mothers had a history of substance use disorders and received follow-up care for addiction. It is hypothesized that women with a history of psychiatric treatment may be more likely to be referred to MBU for PPD.

Moreover, the majority of mothers had an anxiety-related comorbidity, which is consistent with previous research [13,28]. Our study also found a similar prevalence of anxiety among mothers with PPD as a French national study, which reported a prevalence of 83.2% in 2023 [40].

Mothers with bipolar disorder had a greater history of suicide attempts than non-bipolar mothers, which is consistent with the literature [41].

### 4.3. Outcomes Discussion

#### 4.3.1. Primary Outcome

The main finding is a significant decrease in both global DQ and partial DQ for sociability and posture in our population compared to the general population, without reaching pathological values. Indeed, none of the 40 babies had a global developmental delay. These values indicate a trend towards a very early drop in DQ, which requires special care, monitoring, and support. The observed weakness in overall DQ in our cohort is consistent with the literature [20].

Therefore, we can hypothesize that the lack of delay in the 6-month-old infants in our study could be due to the support of mother–baby care provided in the MBU, which could safeguard the child’s development while preserving the parents’ role in the child’s daily care. Nurses can support or replace the mother in the continuity of the child’s daily care. They can also adopt a more reserved attitude but remain available and present to care for the babies.

Infants of mothers with PPD tend to avoid eye contact by frequently turning their heads, showing reduced expression of positive affect and reduced psychomotor activity, which is used to distance themselves from interactional failures [42]. The relative weakness in sociability exhibited by our cohort is also consistent with previous literature [43,44,45].

The postural DQ of our population was found to be significantly lower than that of the general population, despite the exclusion of infants with pathologies that could affect the development of postural tone. Ajuriaguerra in 1974 has described the tonic-emotional dialog, which is the body-to-body exchange between the infant and the caregiver. The preverbal infants express their emotions through tonic dialog, from a tension–relaxation perspective. PPD can affect the mother’s motor skills, muscle tone, and even the way she carries her baby. This, in turn, can impact the baby’s tonic responses [46], which may explain the moderate decrease in postural DQ scores in this population.

Other studies have also demonstrated the impact of depression on infant psychomotor development [38,47,48]. Postural development (which can be likened to gross motor skills) develops earlier and faster than oculomotor coordination (which can be likened to fine motor skills). This temporal evolution may explain the inconsistent results of studies evaluating the effect of PPD on motor development at different ages.

Partial DQ coordination was reduced in infants of mothers with postpartum depression, with a result very close to statistical significance. We can therefore hypothesize that a larger sample could result in a statistically significant decrease in partial DQ coordination.

Our study found no significant change in the partial DQ ’language’ of 6-month-old infants whose mothers had PPD. Previous research has shown that mothers with mood disorders tend to use less language [49] and exhibit reduced voice modulation when addressing their child [50,51,52]. Kaplan et al. [53] demonstrated a significant negative correlation between maternal sadness and expressive communication subscores on the Bayley Test. Other studies have also found that the duration of PPD is associated with a negative impact on the child’s language [54,55,56]. In addition, in the Brunet–Lézine test, the language partial DQ at 6 months is measured partly on the items collected during the parent interview and not exclusively on items observed by the examiner [26], which may explain why the language DQ is not reduced. In fact, we have to take into account the subjectivity of the parents, who are often more optimistic than realistic, and have difficulty admitting a delay or a disorder in their child [57,58]. Furthermore, the authors who found impaired communication in the infants of depressed mothers conducted their study when the children were at least one year old. Therefore, it is possible that our assessment at 6 months of age was too early to detect a significant change in the partial DQ language. This prompts us to continue with assessments at 12 and 18 months. In addition, as mentioned above, the care provided in the MBU may have mitigated the effects of maternal PPD on the child’s communication.

#### 4.3.2. Secondary Outcome

PPD occurring within the context of bipolar disorder has been found to negatively affect all of the children’s developmental quotients compared to children of depressed mothers without bipolarity. However, probably due to lack of statistical power associated with small population size, only the partial postural development quotient was significantly lower. This trend can be attributed to the more severe depressive symptoms of women with bipolar disorder, which have a greater impact on the mother–baby relationship [59].

Regarding the specific impact of bipolar depression on the child’s postural quotient, a link can be made with the findings of Mitchell et al. [60]. They compared the clinical features of bipolar depression with those of non-bipolar depression outside the postpartum context. They found that bipolar patients were less emotionally reactive, had a greater delay in verbal responses, and showed greater psychomotor retardation [29,60]. In the postpartum period, these symptoms can affect the mother’s tonus, rhythm, and mimicry, which may disrupt the tonic-emotional dialog and thus affect the baby’s tonus. This effect could explain the particular impact on the postural partial DQ.

In addition, dyadic coordination disorders have been identified in bipolar depression [30], which may further impair tonic-postural dialog. During the first year, the main dyadic challenge for mothers and infants is to “find” each other and share a positive “rhythmic dance” [61]. Poor dyadic coordination can have a negative impact on child development, as these are essential building blocks for the development of infant social skills and emotion regulation [62,63].

Furthermore, in bipolar disorder, inter-episodic sub-syndromic symptoms have been shown to impair social functioning [29], and may expose infants to chronic pathological interactions with their mothers. This may also explain the particular effect of bipolar depression.

### 4.4. Biases, Limitations, and Strengths of the Study

Potential ranking bias should be considered. It has been shown that psychological support for mothers can lead to significant improvements in the mother–child relationship [64]. In our sample, depressed mothers received psychiatric care before child development was assessed. In our unit, infants were supervised by caregivers who provided appropriate stimulation and attention to the child’s needs, which may help to compensate for maternal withdrawal. It is therefore possible that the treatment provided helped to reduce the developmental impact of PPD. Additionally, the exclusion of mothers presenting a high risk of suicide or infanticide due to the characteristics of our MBU likely led to the exclusion of the most severe PPD cases and potential selection bias.

Some limitations deserve acknowledgment.

First, the sample of infants was relatively small, which reduced statistical power.

Second, the study was conducted at a single center. However, it was a referral center located in the second largest city in France, which serves a large region in the southeast of the country. In addition, the mother–baby relationship care provided in the Marseille unit was consistent with that provided in other units in France [65]. It should be noted that our study took place in a Western European cultural context, which may limit the generalizability of the results. Therefore, the study should be replicated in other cultural contexts.

Another limitation of the study was the inability to stratify women by socioeconomic level due to a lack of information. According to Stein, mothers suffering from PPD with a higher socioeconomic status tend to provide better care for their babies [8]. A potential confounding factor is therefore not analyzed.

Moreover, we conducted a descriptive study that allowed us to hypothesize about the association between PPD and worse scores in DQ, but did not prove a causal relationship. Therefore, etiologic studies should be conducted.

The strength of our study lies in the objective assessment of the babies by a blind operator using a validated and parameterized scale. This reduces the measurement bias associated with raters’ subjectivity.

## 5. Conclusions

This study examined the development of 6-month-old infants whose mothers had severe PPD requiring joint hospitalization. The findings indicate a decrease in the overall DQ, but no evidence of developmental delay. In addition, there were significant decreases in the partial DQs for sociability and posture. It is important to note that these results need to be confirmed by multicenter studies.

Although preliminary, our results suggest a fragility of sociability and an impact on postural development in infants whose mothers have PPD. This highlights the need for a more objective measure than clinical daily observation of a baby’s behavior. This assessment with validated tools should be part of a systematic evaluation to identify the baby’s strengths and weaknesses, as well as early signs of developmental delays or disorders. Regular reassessment can help ensure that the child is responding well to the dyad’s care. However, if a child continues to fall behind in one or more areas despite the care provided, a diagnostic evaluation of the child and/or a therapeutic adjustment are suggested.

In addition, this study raises awareness among healthcare providers and child care professionals of the very early impact of PPD symptoms on infant development. It is important to note that the first year of life is the period of greatest brain plasticity. Screening children at an early age with specific tools allows for earlier intervention, which would positively impact on their developmental trajectory. MBUs, complemented by mobile perinatal teams, are an early intervention modality that treats both maternal postpartum depression and mother–baby interactions through care that enables the mother to reinvest positively in her relationship with her baby and supports the baby’s development.

## Figures and Tables

**Table 1:** Medical and socio-demographic characteristics of mothers.

Variable	Value	*n*
age		
mean (SD)	34 (6)	
median (IQR)	33 (8.2)	
min–max	20–46	
first child		
yes	73.7%	28
no	26.3%	10
marital status		
in a relationship	92.1%	35
separated	7.9%	3
history of drug use		
yes	15.8%	6
no	84.2%	32
history of depression		
yes	55.3%	21
no	44.7%	17
history of suicide attempt		
yes	10%	4
no	90%	36
history of eating disorder		
yes	10.5%	4
no	89.5%	34
other previous history		
burn-out	2.6%	1
periconceptional mourning	2.6%	1
mental disability	2.6%	1
bipolarity comorbidity		
yes	26.3%	10
no	73.7%	28
anxiety comorbidity		
yes	78.9%	30
no	21.1%	8

SD: Standard Deviation; IQR: Interquartile Range.

**Table 2:** Medical and sociodemographic characteristics of mothers according to the presence or absence of bipolarity.

Variable	Non-Bipolar (*n* = 28)	*n*	Bipolar (*n* = 10)	*n*	*p*-Value
age					
mean (SD)	33.9 (6.1)		34.3 (6)		0.858
median (IQR)	33 (8.8)		33 (5.5)		
min–max	20–45		27–46		
first child					0.699
yes	71.4%	20	80%	8	
no	28.6%	8	20%	2	
history of drug use					0.310
yes	10.7%	3	30%	3	
no	89.2%	25	70%	7	
history of depression					**0.012**
yes	42.9%	12	90%	9	
no	57.1%	16	10%	1	
history of suicide attempt					**0.048**
yes	3.6%	1	30%	3	
no	96.4%	27	70%	7	
history of eating disorder					0.556
yes	14.3%	4	0%	0	
no	85.7%	24	100%	10	
other previous history					1
burn out	3.6%	1	0%	0	
periconceptional mourning	3.6%	1	0%	0	
mental disability	3.6%	1	0%	0	
marital status					1.000
in a relationship	92.9%	26	90%	9	
separated	7.1%	2	10%	1	
anxiety comorbidity					0.411
yes	82.1%	23	70%	7	
no	17.9%	5	30%	3	

**Bold** indicates *p* < 0.05.

**Table 3:** Development quotients results (overall and partial).

Domain	Statistics	Values	*p*-Value	ND
	mean (SD)	96.2 (8.9)	**<0.001**	no
Posture	median [IQR]	96 [9.2]		
	min–max	74–129		
	mean (SD)	97.1 (10)	0.058	no
Coordination	median [IQR]	98 [10.2]		
	min–max	58–120		
	mean (SD)	101.2 (8.9)	0.3973	yes
Language	median [IQR]	100.5 [10.5]		
	min–max	83–115		
	mean (SD)	94 (9.6)	**<0.001**	yes
Sociability	median [IQR]	96 [11.5]		
	min–max	70–118		
	mean (SD)	96.9 (6.7)	**<0.001**	no
Global	median [IQR]	96.5 (6)		
	min–max	78–119		

SD: Standard Deviation; IQR: Interquartile Range; ND: normal distribution (assessed by the Shapiro–Wilk test); **Bold** indicates *p* < 0.05.

**Table 4:** Comparison of infant’s global and partial developmental quotients according to whether or not the mother has bipolar disorder.

DQ	Parameters	Non-Bipolar	Bipolar	*p*-Value	ND
Posture	mean (SD)	98.1 (8)	90.2 (9.1)	**0.0295**	no
	median [IQR]	98 [5.8]	89.5 [12.5]		
	min–max	86–129	74–103		
Coordination	mean (SD)	98.1 (8.3)	94.1 (14.1)	0.7307	no
	median [IQR]	99 (10)	97 [10.8]		
	min–max	79–120	58–106		
Language	mean (SD)	101.8 (8.2)	99.4 (11)	0.5376	yes
	median [IQR]	100.5 (10)	102.5 [16.8]		
	min–max	83–115	83–114		
Sociability	mean (SD)	94.7 (9.4)	92.1 (10.5)	0.4994	yes
	median [IQR]	96 [13.2]	94.5 [7.5]		
	min–max	79–118	70–105		
Global	mean (SD)	98.2 (5.7)	93 (8.2)	0.2399	no
	median [IQR]	96 (6)	97 [11.5]		
	min–max	90–119	78–100		

SD: Standard Deviation; IQR: Interquartile Range; ND: normal distribution (defined by the Shapiro–Wilk test). **Bold** indicates *p* < 0.05.

## Data Availability

All data generated for this study are available from the corresponding author upon reasonable request. The data are not publicly available to protect the data privacy and restrict unauthorized use.
